# NIR-PIT: Will it become a standard cancer treatment?

**DOI:** 10.3389/fonc.2022.1008162

**Published:** 2022-09-16

**Authors:** Aki Furusawa, Peter L. Choyke, Hisataka Kobayashi

**Affiliations:** Molecular Imaging Branch, Center for Cancer Research, National Cancer Institute, National Institutes of Health, Bethesda, MD, United States

**Keywords:** near infrared photoimmunotherapy, cancer, host immunity, theranostic molecular imaging, standard therapy, NIR-PIT

## Introduction

Near-infrared photoimmunotherapy (NIR-PIT) is a newly developed technology that selectively destroys targeted cells (1, 2). The two major elements of this therapy are a conjugate of a monoclonal antibody (mAb) and the phthalocyanine-based photoabsorber dye IRDye700DX (IR700) and the application of near-infrared (NIR) light to the target. The mAb-photoabrsorber conjugate (AbPC) is injected into the patients’ body where it accumulates at the targeted site. Upon NIR irradiation, a ligand release reaction takes place in the IR700 molecule making the dye extremely hydrophobic and causing aggregation of the AbPC and cell membrane (3). This causes damage in the cell membranes of target cells, leading to necrotic cell death (4). Theoretically, NIR-PIT can destroy any cell as long as the AbPC can bind in sufficient quantities to result in cell damage. When antibodies against a cell surface antigen on cancer cells are used, cancer cells are selectively killed in a process known as cancer cell targeted-NIR-PIT. NIR-PIT can also destroy immune-suppressor cells in the tumor microenvironment by targeting surface antigens on immune suppressor cells (5). In this article, we mainly discuss cancer-cell targeted NIR-PIT but immune-cell-targeted NIR-PIT is of growing importance in extending the benefits of NIR-PIT. Cell destruction only occurs in the cells to which the AbPCs are bound, thus neighboring cells not expressing the target are typically unharmed. This is a huge advantage in treating tumors, especially when the tumor is located in complex parts of the body, close to vital structures such as the head and neck. In this setting, NIR-PIT can provide a minimally invasive but highly selective treatment.

Currently, EGFR-targeted NIR-PIT for advanced head and neck cancers is approved for clinical use in Japan, and phase III clinical trials are ongoing worldwide. EGFR is one of the most commonly expressed cancer cell surface antigens, thus EGFR-targeted NIR-PIT is applicable to many other types of patients with EGFR-expressing tumors. However, for patients whose cancer cells don’t express EGFR, an alternative target is necessary.

NIR-PIT has been shown to be effective and safe. NIR-PIT is cell-specific and locally restricted, therefore, less-invasive compared to surgical resection. Also, relatively inexpensive equipment is required to perform NIR-PIT including a NIR laser light source and light application probes which typically cost less than 100k. Thus, NIR-PIT has the potential to become a major new tool in the fight against cancer.

In this article, we discuss what steps are necessary to make NIR-PIT a new standard therapy for cancer.

## Increasing the number of NIR-PIT targets

### Wide variety of NIR-PIT targets facilitate personalized cancer treatment

So far, only EGFR-targeted PIT has been approved for clinical use. However, every cancer has a different antigen expression profile and not all tumors express EGFR. To make this therapy widely applicable to various cancers, it is necessary to test more cancer cell targets and expand the inventory of targets of NIR-PIT. Once more targets are available, more personalized treatment will be possible. For example, we can perform a preliminary biopsy to examine the tumor’s antigen expression profile and then decide which target to use. Various targets are under pre-clinical investigation, and studies targeting antigens such as CD44, PSMA, and podoplanin showed promising results (6). Clinical studies for NIR-PIT targeting these antigens are anticipated.

### Be mindful of the expression in non-cancer cells

When choosing the target antigen for cancer cell-targeted NIR-PIT, the target has to be highly expressed in cancer cells. However, expressions in non-cancer cells also have to be taken into account. If the antigen is also expressed in non-cancer cells, these cells will be killed along with cancer cells. Thus, we need to be mindful of the antigen expression in the treatment region so that no significant damage is done to other vital tissue around the tumor. Also, the target antigen should not be expressed in immune cells, especially dendritic cells and T cells, as explained in the later section.

### Be mindful of tumor heterogeneity

Cancer cells in a tumor are often heterogeneous; different antigens are expressed in each distinct pattern. Targeting more aggressive or stem-like cancer cell populations is ideal for eradicating tumors efficiently. When multiple antigens are expressed like a mosaic, it is possible to target multiple antigens simultaneously.

### Previously approved antibody medicines as NIR-PIT agent

To expand the inventory of cancer cell-targeted NIR-PIT targets, there have to be suitable antibodies that bind to the cancer cell surface. Fortunately, there are many available clinical antibodies already approved and their number and diversity are expanding every year (7, 8). Some of these antibodies bind antigens on cancer cells and can be utilized as the material of AbPC to target cancer cells. There also are antibodies that failed to show efficacy in clinical trials as monoclonal antibody therapy (9, 10) that can be repurposed as NIR-PIT agents in order to breathe new life into them.

## To harness NIR-PIT-induced anti-cancer immune activation

### Anti-cancer immune activation after NIR-PIT

The most well-known feature of NIR-PIT is direct, selective cancer cell killing. However, there is another extremely valuable but often overlooked feature that is prompted by cancer cell killing; anti-cancer immune activation following NIR-PIT. When cancer cells are killed after NIR light irradiation, the cell contents are released and are captured by antigen-presenting cells (APCs) such as dendritic cells (DCs) (11). APCs then migrate to lymph nodes (LNs) where T cells are activated. Thus, NIR-PIT induces immunogenic cell death. Such immune activation cannot be induced after other conventional treatments such as surgical resection or chemotherapy. The activated T cells migrate throughout the entire body, creating an anti-cancer immune response that works systemically, even in non-NIR-irradiated tissues. Indeed, when only one of the tumors in the bilateral tumor model was treated by cancer-cell targeted NIR-PIT, not only the treated tumor regressed but the untreated side tumor also shrunk; this phenomenon is called the abscopal effect (11). Such immune activation occurs against various antigens that are expressed in target cancer cells (11), so NIR-PIT can be effective even against cancer cells that do not express the original NIR-PIT target. Such an effect is called antigen spreading. Once immune memory is established, the anti-cancer immune surveillance can last a long time. For example, once the tumor is eradicated after NIR-PIT, the same cancer model could not be re-introduced into the animal due to its acquired immunity against that tumor strain.

Therefore, efficiently inducing anti-cancer immune activation by NIR-PIT greatly benefits patients not only by destroying primary tumors but also by preventing recurrence and metastasis.

### Need for research on immunocompetent animal models

Many studies test the efficacy of NIR-PIT in mouse xenograft models that have human cancer cells in immune-deficient mice. Xenograft models are useful to test the cell-killing efficacy of NIR-PIT against human cells, however, studies in xenograft models alone are not enough to assess the overall treatment efficacy of NIR-PIT.

One reason is that, in this case, the host mice are immune-deficient. For example, in T cell-lacking athymic nude mice, it is impossible to study T cell-mediated anti-cancer immunity. Anti-cancer immunity induced by NIR-PIT is thought to be T cell dependent.

Another reason that xenograft studies are suboptimal is that the cancer cells and host cells are from different species therefore they are not bound by the same antibody. NIR-PIT can destroy non-cancer cells as well if the target antigen is expressed. To assess such effects, cancer cells and host cells need to be from the same species. So, it is ideal to test the given NIR-PIT in syngeneic or transgenic mouse models to see the effect on other non-cancer cells in the treatment site (12, 13).

Upon choosing a cancer cell target antigen, we have to be mindful of the antigen’s expression in immune cells. APCs including DCs are critical to the immune response because those cells capture the antigen released from cancer cells undergoing immunogenic cell death and migrate to LNs where they activate T cells. Indeed, when CD29-targeted PIT and CD44-targeted PIT were compared in the same tumor model, CD29-targeted PIT induced a more robust immune activation. This was thought to be because CD29 has minimal expression in immune cells including DC, and CD29-targeted NIR-PIT does not kill those cells, unlike CD44-targeted PIT (14). Such research has to be done in immune-competent animal models, not in immune-deficient mice.

### Combination therapies of NIR-PIT and immune-activating agents

Anti-cancer immune activation induced by NIR-PIT can be enhanced when the therapy is combined with other immune-activating agents. So far, the combination of NIR-PIT with immune checkpoint inhibitors such as anti-PD-1 and anti-CTLA-4 mAbs (11, 15), or with activation cytokines such as IL-15 have been tested and shown effective in augmenting the efficacy of cancer-cell targeted PIT by enhancing the anti-cancer immune activation (16). These agents modulate different steps of immune activation, so further research regarding dosing and timing will be needed to maximize the effect of these combination therapies. There are also other immunomodulators and other cell-surface target antigens for immune suppressor cells (17, 18) that can be tested in combination with NIR-PIT.

Thus, immune-suppressor cell targeted NIR-PIT has been shown to be effective in augmenting anti-cancer immunity, and once these therapies are clinically approved, the combination therapy with cancer cell targeted NIR-PIT, that has been demonstrated to be effective in preclinical studies (19, 20), should be tested.

## Rationally monitoring therapy and therapeutic outcome of NIR-PIT with molecular imaging

### Completing NIR-PIT therapy during procedure

Since cytotoxicity of AbPC-bound target cells induced by NIR-PIT is based on photo-induced ligand release reaction of IR700, one means of assessing NIR-PIT during light exposure is to measure IR700 fluorescence during the procedure. IR700 loses its fluorescence signal immediately after the ligands are released from the phthalocyanine ring of IR700 (3). Therefore, we have demonstrated that fluorescence loss of IR700 is closely related to the effectiveness of the therapy (21). In order to practically apply the IR700 fluorescence monitoring in the clinic, we employed a commercially-available fluorescence camera system, Lightvision™ (Shimadzu Inc, Kyoto, Japan) which was originally designed for indocyanine green and has been approved for sentinel lymph node detection and several fluorescence-guided surgeries by the US FDA and PMDA in Japan. Detectable emission wavelength of the camera system ranges between ~830 to ~850 nm which is in the tail emission of IR700 with minimal biological autofluorescence and without spilling out of strong therapeutic excitation light (150 mW/cm^2^) and the room light in surgical suites. IR700 fluorescence decay was monitored in real-time with the camera system during experimental NIR-PIT in mice, and correlated well with the therapeutic outcome (21). From the analysis of dynamic decay curves of IR700 fluorescence during NIR-PIT, current light dosing that is typically used for NIR-PIT can be safely reduced without compromising treatment effectiveness. In a current clinical trial of NIR-PIT in newly diagnosed head and neck cancers, we are using a fluorescence camera during application of NIR light to monitor the therapeutic progress of NIR-PIT in order to establish the optimal dose of NIR light exposure.

### Acute therapeutic effects of NIR-PIT

Since NIR-PIT induces immunogenic cell death in targeted cancer cells, rapid changes in metabolism of cancer cells after NIR-PIT can be observed. However, tumor volume changes much more slowly with gradual changes seen over several days after NIR-PIT because post treatment edema generally adds some volume to the tumor mass even while the cells are dying. Therefore, metabolic molecular imaging such as ^18^F-FDG-PET is an appropriate tool to monitor acute therapeutic effects of NIR-PIT. In a mouse model, we demonstrated that uptake of 18F-FDG decreased more than 90% 1.25 h after NIR-PIT despite the absence of changes in tumor size and shape (22). Therefore, we are now using early ^18^F-FDG-PET as a monitoring method of the acute therapeutic effects of NIR-PIT to target cancer cells. Rapidly activated host immunity in tumor beds and associated edema around tumor beds after NIR-PIT, however, might increase the uptake of 18F-FDG limiting the utility of this test in humans with intact immune systems.

## Conclusion

**Figure 1:**
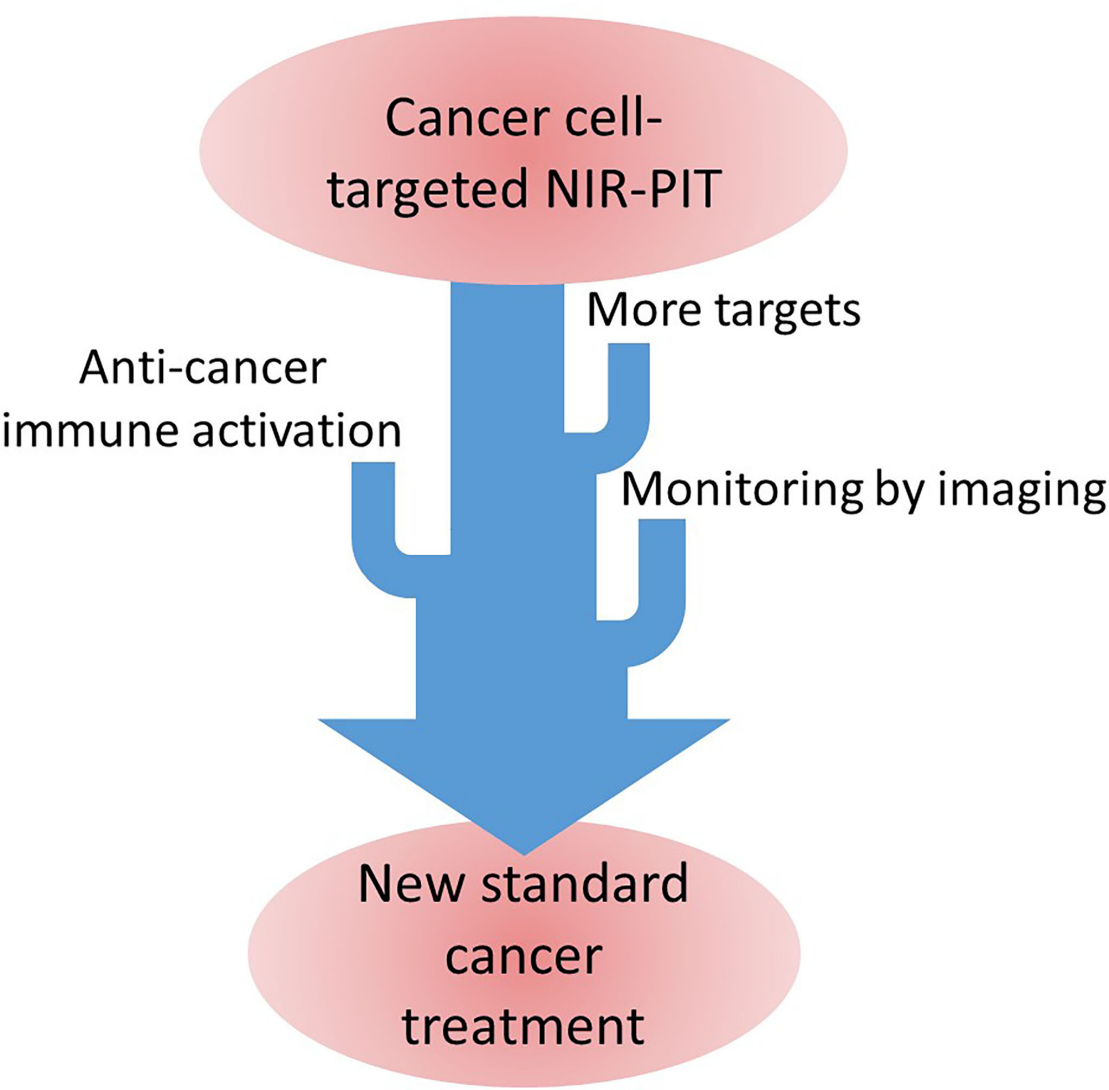
Diagram of our current progress plan of the NIR-PIT toward the standard cancer treatment.

Clinical application of NIR-PIT has just started but applicable cancer types are still limited. When more studies on (1); more NIR-PIT targets, (2), efficient immune activation induced by NIR-PIT, and (3) imaging methods for treatment monitoring, NIR-PIT can become a standard treatment for cancer. (**Figure 1**) NIR-PIT is a less invasive, highly effective treatment that can be combined with other standard cancer treatments. Making NIR-PIT widely applicable would greatly improve cancer patients’ outcome including QOL.

## Author contributions

AF planned and wrote the review article. PC edited and finalized the review article. HK planned, wrote, edited, and supervised the review article. All authors contributed to the article and approved the submitted version.

## Funding

This review was supported by the Intramural Research Program of the NIH, National Cancer Institute, Center for Cancer Research (ZIA BC 011513).

## Conflict of interest

The authors declare that the research was conducted in the absence of any commercial or financial relationships that could be construed as a potential conflict of interest.

## Publisher’s note

All claims expressed in this article are solely those of the authors and do not necessarily represent those of their affiliated organizations, or those of the publisher, the editors and the reviewers. Any product that may be evaluated in this article, or claim that may be made by its manufacturer, is not guaranteed or endorsed by the publisher.
